# 

**Published:** 1981

**Authors:** 


### Contents

3 **The Postmortem is not Dead**
N. J. Brown
*Presidential address to Bristol Medico-Chirurgical Society, 8th October 1980*

14 **The Surprises of General Practice**
Philip Radford

17 **Skull X-Ray and CT. Scan in the Management of Head Injuries**
D. J. Tawn

20 **South West Surgeons Club**
Abstracts of papers given at the meeting in Cork, 1st-2nd May 1982

25 **Book Review**
Operative Surgery and Management

26 **Examination Results**

